# Cancer-induced FOXP1 disrupts and reprograms skeletal-muscle circadian transcription in cachexia

**DOI:** 10.1016/j.celrep.2025.115689

**Published:** 2025-05-10

**Authors:** Jeremy B. Ducharme, Daria Neyroud, Martin M. Schonk, Miguel A. Gutierrez-Monreal, Zhiguang Huo, Haley O. Tucker, Karyn A. Esser, Sarah M. Judge, Andrew R. Judge

**Affiliations:** 1 Department of Physical Therapy, University of Florida, Gainesville, FL, USA; 2 Myology Institute, University of Florida, Gainesville, FL, USA; 3 University of Florida Health Cancer Center, Gainesville, FL, USA; 4 Department of Physiology and Aging, University of Florida, Gainesville, FL, USA; 5 Department of Biostatistics, University of Florida, Gainesville, FL, USA; 6 Department of Molecular Biosciences and the Institute for Cellular and Molecular Biology, University of Texas at Austin, Austin, TX, USA; 7 Senior author; 8 Lead contact

## Abstract

Cancer cachexia is a debilitating metabolic disorder characterized by involuntary loss of body and muscle mass, leading to increased morbidity and mortality. We previously found that forkhead box P1 (FoxP1) upregulation in skeletal muscle causes muscle wasting and is required for muscle wasting in response to cancer. However, transcriptional networks targeted by FoxP1 in skeletal muscles undergoing cancer-induced wasting remain largely unknown. Here, we identify FoxP1 as a key disruptor of the skeletal-muscle clock in response to cancer that reprograms circadian patterns of gene expression at cachexia onset. Specifically, we show that cancer-induced FoxP1 rewires the skeletal-muscle circadian transcriptome toward pathways associated with muscle wasting and disrupts the temporal patterning of pathways governing glucose, lipid, and oxidative metabolism. These findings thus implicate cancer/disease-specific functions of FOXP1 in the disruption and reprograming of the skeletal-muscle circadian transcriptome, which may contribute to muscle wasting and the development of cachexia.

## In brief

Ducharme et al. investigate the impact and crosstalk between cancer and FoxP1 on the skeletal-muscle circadian transcriptome that mediates time-of-day-dependent biological functions. They reveal cancer/disease-specific functions of FoxP1 in the disruption and reprograming of the skeletal-muscle circadian transcriptome, which may contribute to cancer-induced muscle loss.

## Graphical Abstract

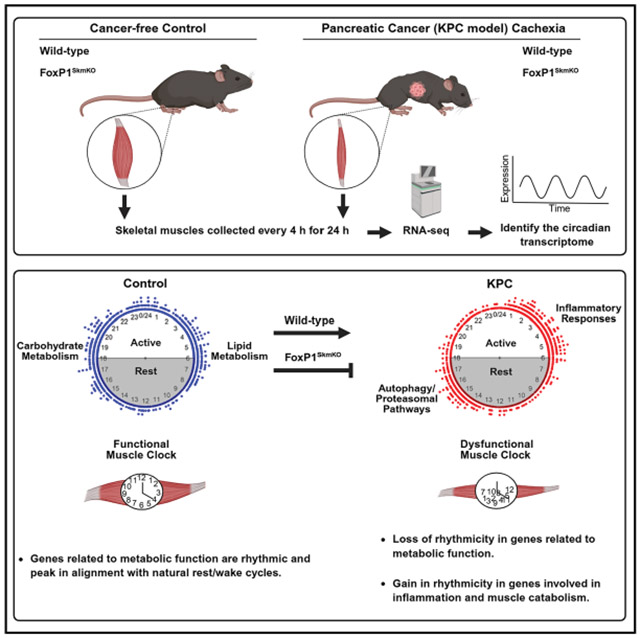

## INTRODUCTION

Involuntary weight loss due to skeletal-muscle wasting, a condition known as cachexia, occurs in response to several chronic diseases, including heart failure, chronic obstructive pulmonary disease, diabetes, AIDS, kidney disease, and cancer.^1^ The prevalence of cachexia is particularly high in certain cancers, such as pancreatic cancer, where the vast majority of patients exhibit cachexia at the time of diagnosis.^2^ Ultimately, cachexia develops in most advanced-stage cancer patients.^2^ Individuals with cancer who develop cachexia suffer from a reduced ability to carry out activities of daily living, a lower tolerance to cancer treatment, a diminished therapeutic response to chemotherapy, and increased mortality.^3^

The initiating factors that cause cancer-induced skeletal-muscle wasting are complex and still incompletely defined. However, in general, wasting occurs due to tumor-induced host adaptation triggered by tumor-secreted factors and the host response. In skeletal muscle, this can cause a mismatch between pathways of protein synthesis and protein degradation and thus disrupt protein balance. However, our understanding of the molecular processes regulating these pathways in muscle is still evolving.

To this end, several transcription factors have been implicated in the atrophy process in muscle through their ability to drive the expression of atrophy-related genes. These include nuclear factor κ-B (NF-κB), signal transducer and activator of transcription (STAT), suppressor of mothers against decapentaplegic (SMAD), CCAAT/enhancer-binding protein beta (C/EBPβ), activator protein-1 (AP-1), and forkhead box O (FoxO).^4-10^ With regard to FoxO, there are three family members expressed in skeletal muscle: FoxO1, FoxO3a, and FoxO4. Previous work has shown that overexpression of either FoxO1 or FoxO3a, both of which are increased in atrophying muscles from tumor-bearing mice, is sufficient to cause muscle-fiber atrophy.^5,8^ FoxO1 is also increased in the skeletal muscle of human cancer patients.^11^ Importantly, inhibition of FoxO transcriptional activity in skeletal muscle of tumor-bearing mice has been shown to inhibit muscle-fiber atrophy, demonstrating the requirement of FoxO for the atrophy phenotype.^8^ In previous work, we identified an additional forkhead box family member, forkhead box P1 (FoxP1), as a downstream target of FoxO1 that is increased in skeletal muscle of cachectic tumor-bearing mice and patients with cancer.^12^ We further showed that FoxP1 overexpression in skeletal muscle is sufficient to cause muscle wasting, weakness, and pathological muscle remodeling; that FoxP1 knockdown in muscle attenuates cancer-induced muscle-fiber atrophy in the C26 colorectal cancer model^12^; and that skeletal-muscle-specific deletion of FoxP1 attenuates muscle wasting and weakness in the KPC pancreatic cancer model.^13^ This work highlighted a key role of FoxP1 in the regulation of muscle wasting and provided the foundation for further investigation into its regulatory network in skeletal muscle, including the genes it directly targets.

In the current study, we therefore aimed to investigate the transcriptional networks targeted by FoxP1 in skeletal muscle both in cancer-free conditions and in skeletal muscles undergoing wasting due to pancreatic cancer. Using chromatin immunoprecipitation followed by next-generation sequencing (ChIP-seq), we identified an enrichment of genes central to the core circadian clock system as direct targets of FOXP1 binding. We further found that skeletal-muscle FoxP1 modulates core clock gene expression in response to cancer, including repression of core clock activators and activation of core clock repressors. We next evaluated the extent to which pancreatic cancer impacts circadian patterns of gene expression in skeletal muscle and whether this is mediated via FoxP1. Our findings identify that cancer-induced FoxP1 disrupts and rewires circadian patterns of gene expression in skeletal muscle that may contribute to the development of cachexia. Specifically, we identified that, at a time point corresponding to cachexia initiation,^14,15^ pancreatic cancer causes a significant loss of rhythmicity in gene programs that normally exhibit circadian expression in skeletal muscle, including those associated with carbohydrate, lipid, and oxidative metabolism, while causing a gain in rhythmicity in gene programs linked to muscle wasting, including autophagy, the ubiquitin-proteasome system, and inflammation. These effects largely occurred in a FoxP1-dependent manner, supporting FoxP1 as a key disruptor of circadian programs in skeletal muscle in response to cancer that may contribute to muscle loss.

## RESULTS

### FOXP1 DNA-binding sites in skeletal muscle

**Figure 1.**
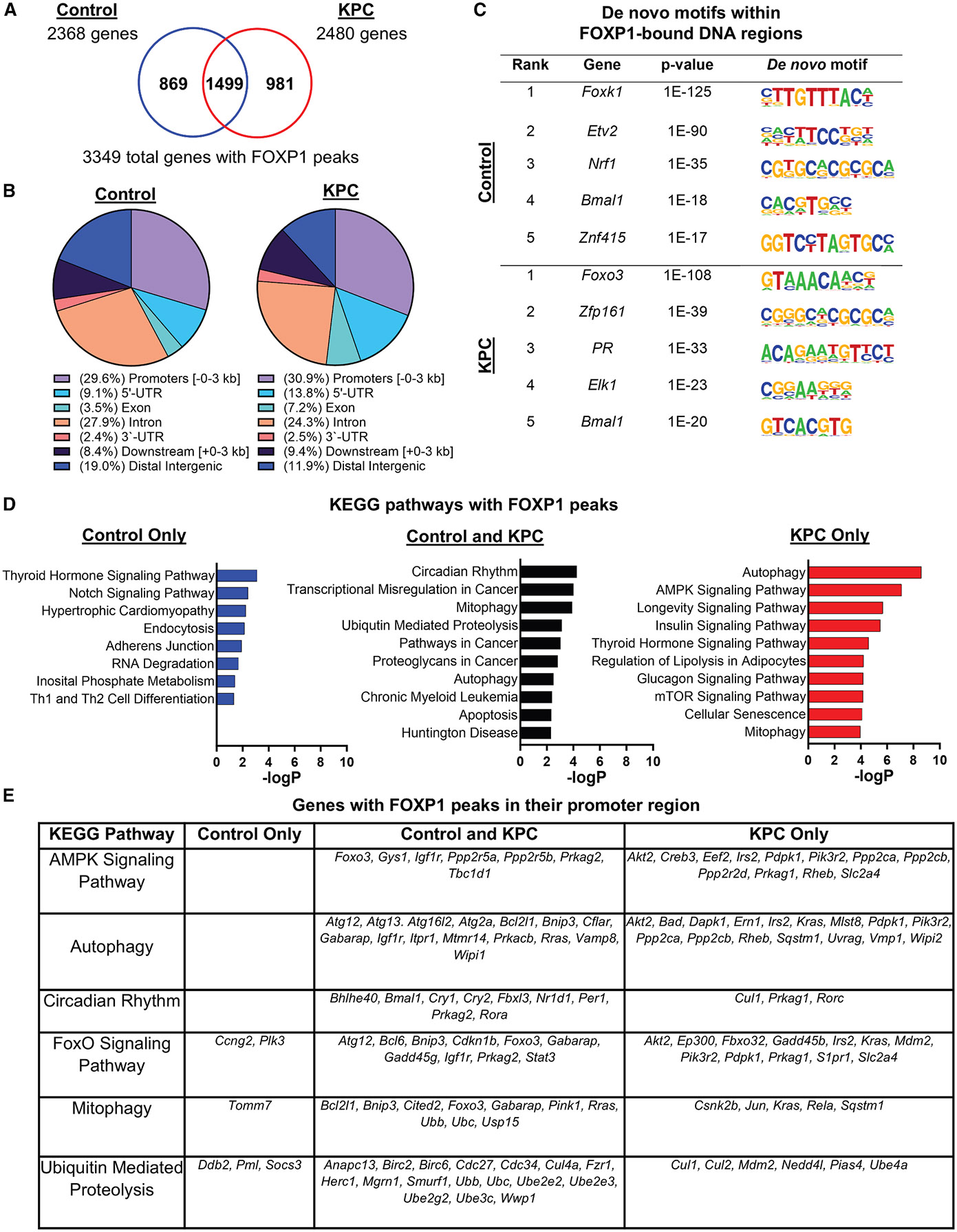
FOXP1 DNA-binding sites within skeletal muscle of mice with and without KPC pancreatic tumors (A) Overlap of genes with FOXP1-binding sites between conditions. (B) Average distribution of FOXP1-binding sites by location in genes. (C) Representative top-ranking enriched *de novo* motifs found in chromatin sites occupied by FoxP1 within each condition. (D) Top-enriched KEGG pathways of genes with FOXP1 peaks that are unique to control mice, KPC mice, or are expressed in both conditions (*p* < 0.05). (E) Genes with FOXP1 binding in their promoter region annotating to enriched KEGG pathways of interest. *n* = 2 mice/group. See also Tables S1 and S5.

To identify gene regulatory regions directly bound by endogenous FOXP1 in skeletal muscle during control (cancer-free) and tumor-bearing conditions, we mapped FOXP1-binding events using ChIP-seq in the gastrocnemius-plantaris muscle complex from mice with and without KPC pancreatic tumors at a time point corresponding to cachexia onset.^14,15^ Binding events were identified using model-based analysis for ChIP-seq (MACS),^16^ which discovered 4,075 FOXP1-binding sites on 2,368 genes in skeletal muscle from control mice and 5,639 FOXP1-binding sites on 2,480 genes in skeletal muscle from KPC mice (Figure 1A). An overlap of these conditions revealed 981 unique genes with FOXP1-binding sites in KPC mice only, 869 genes in control mice only, and 1,499 genes shared by both control and KPC mice (Table S1). The greatest percentage of FOXP1-binding sites were observed in promoter (~30%) and intronic (~25%) regions, followed by distal intergenic regions (~12%–20%, i.e., DNA locations between genes) and 5′ untranslated regions (~9%–14%) (Figure 1B). The top-ranking *de novo* motif within FOXP1-bound DNA regions identified via HOMER in both conditions was a consensus motif that aligned with the forkhead motif (Figure 1C), thereby affirming the specificity of FOXP1 binding to DNA regions that contain this motif. Further examination of the top five overrepresented *de novo* motifs enriched in target sequences bound by FOXP1 in each condition revealed a strong enrichment to key members of the E-twenty-six (ETS) family of transcription factors, and transcription factors that regulate mitochondrial biogenesis (e.g., nuclear respiratory factor 1 [NRF1]) and circadian transcriptional programs (e.g., brain and muscle arnt-like 1 [BMAL1]).

To further assess the regulatory role of FoxP1 in skeletal muscle, we employed EnrichR to analyze genes with FOXP1-binding sites found in control mice only, KPC mice only, or both control and KPC mice to determine the most highly enriched Kyoto Encyclopedia of Genes and Genomes (KEGG) pathways (Figures 1D and 1E; Table S1). FOXP1-bound genes in both control and KPC mice were enriched for pathways involved in circadian rhythm, including *Bmal1, Per1, Cry1,* and *Cry2,* which are components of the core clock that regulate time-of-day-dependent biological functions. Each of these core clock genes demonstrated FOXP1 binding within their promoter regions. Also enriched among FOXP1-bound genes were pathways related to muscle protein quality control, including mitophagy (e.g., *Bcl2l1, Bnip3,* and *Pink1*), autophagy (e.g., *Gabarap, Atg12,* and *Atg2a*), and ubiquitin-mediated proteolysis (e.g., *Ubc, Herc1,* and *Cdc34*). In contrast, various genes related to metabolic regulation were bound by FOXP1 in muscle of KPC mice only, including those annotating to insulin signaling (e.g., *Irs2, Slc2a4/Glut4,* and *Mtor*), regulation of lipolysis in adipocytes (e.g., *Npr1, Mgll,* and *Pnpla2*), and glucagon signaling (e.g., *Creb3, Crtc2,* and *FoxO1*).

### Skeletal-muscle FOXP1 represses core clock gene expression in response to cancer

**Figure 2.**
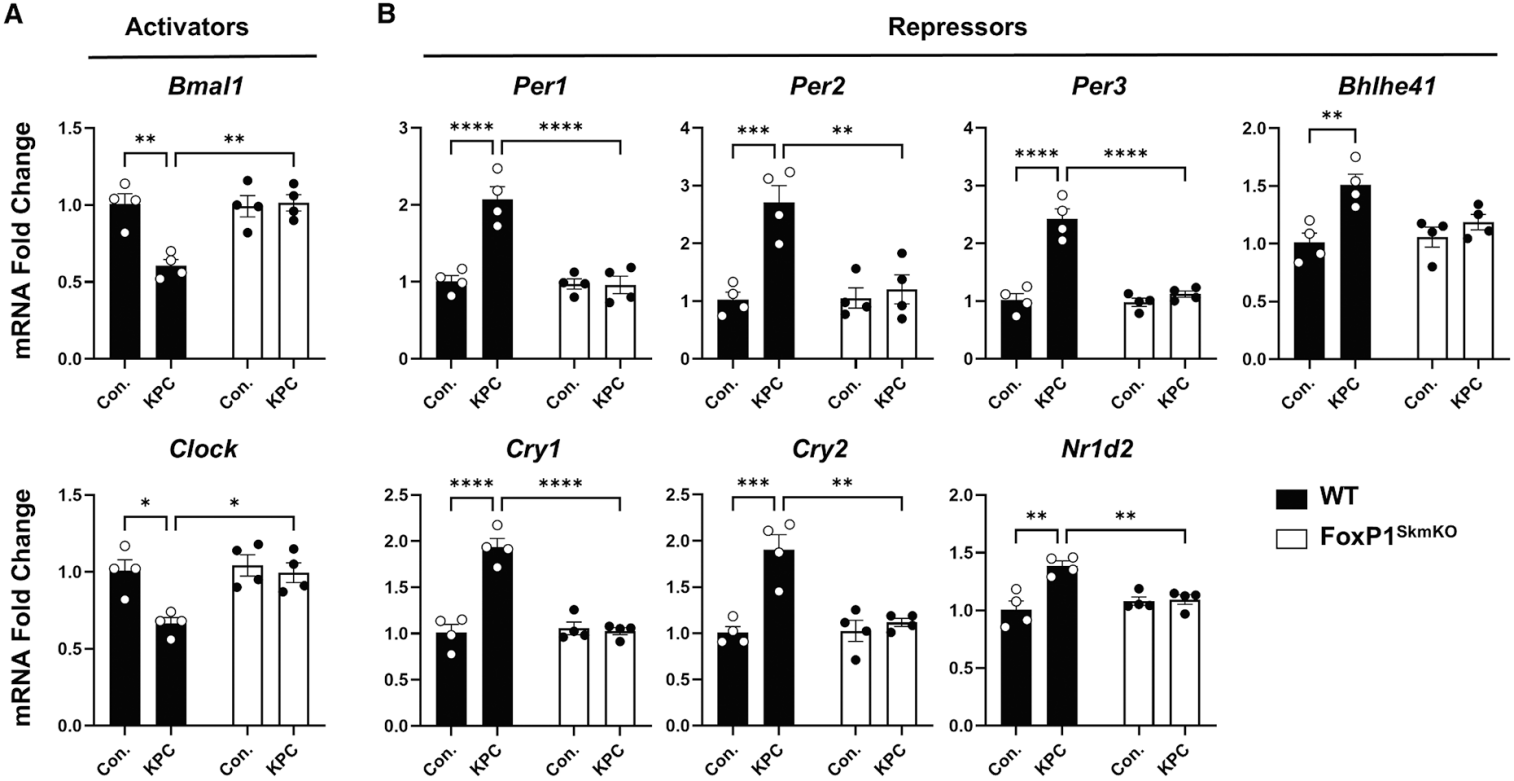
Skeletal-muscle FOXP1 represses core clock gene expression in response to KPC pancreatic tumors Gene expression changes of select components of the core circadian clock activators (A) and repressors (B) were evaluated using RT-qPCR analyses. Differences between groups were assessed using two-way ANOVA with Holm-Sidak’s *post hoc* test for multiple comparisons (*p < 0.05, **p < 0.01, ***p < 0.001, ****p < 0.0001). Data represent individual responses along with mean ± standard error. *n* = 4 mice/condition/genotype.

Using skeletal muscles harvested from control and KPC tumor-bearing wild-type (WT) mice and mice with skeletal-muscle-specific knockout of FoxP1 (FoxP1^SkmKO^), we further performed RT-qPCR analysis to determine whether select core clock genes directly bound by FOXP1 within their promoters are dysregulated in a FoxP1-dependent manner in response to cancer. We found that, in WT mice, but not FoxP1^SkmKO^ mice, pancreatic cancer caused a substantial disruption to the gene expression of core clock components, including repression of *Bmal1,* which is part of the activating arm of the clock, and activation of *Per1, Cry1,* and *Cry2,* which are part of the repressive arm of the clock (Figure 2). We further found that additional core clock genes that are part of the repressive arm were increased in WT KPC but not FoxP1^SkmKO^ KPC mice, including *Per2, Per3, Nr1d2,* and *Bhlhe41.* The identification of FOXP1 as a modulator of these core clock genes in response to cancer is of potential significance, as disruptions to the clock may perturb normal time-of-day-dependent oscillations in rhythmically expressed genes that are critical for muscle homeostasis. In this regard, disruption to the skeletal muscle clock through muscle-specific deletion of *Bmal1* significantly alters transcriptional programs governing metabolic processes in muscle and is sufficient to induce muscle atrophy and weakness.^17-21^

### Circadian patterns of gene expression are disrupted in skeletal muscle in response to cancer

**Figure 3.**
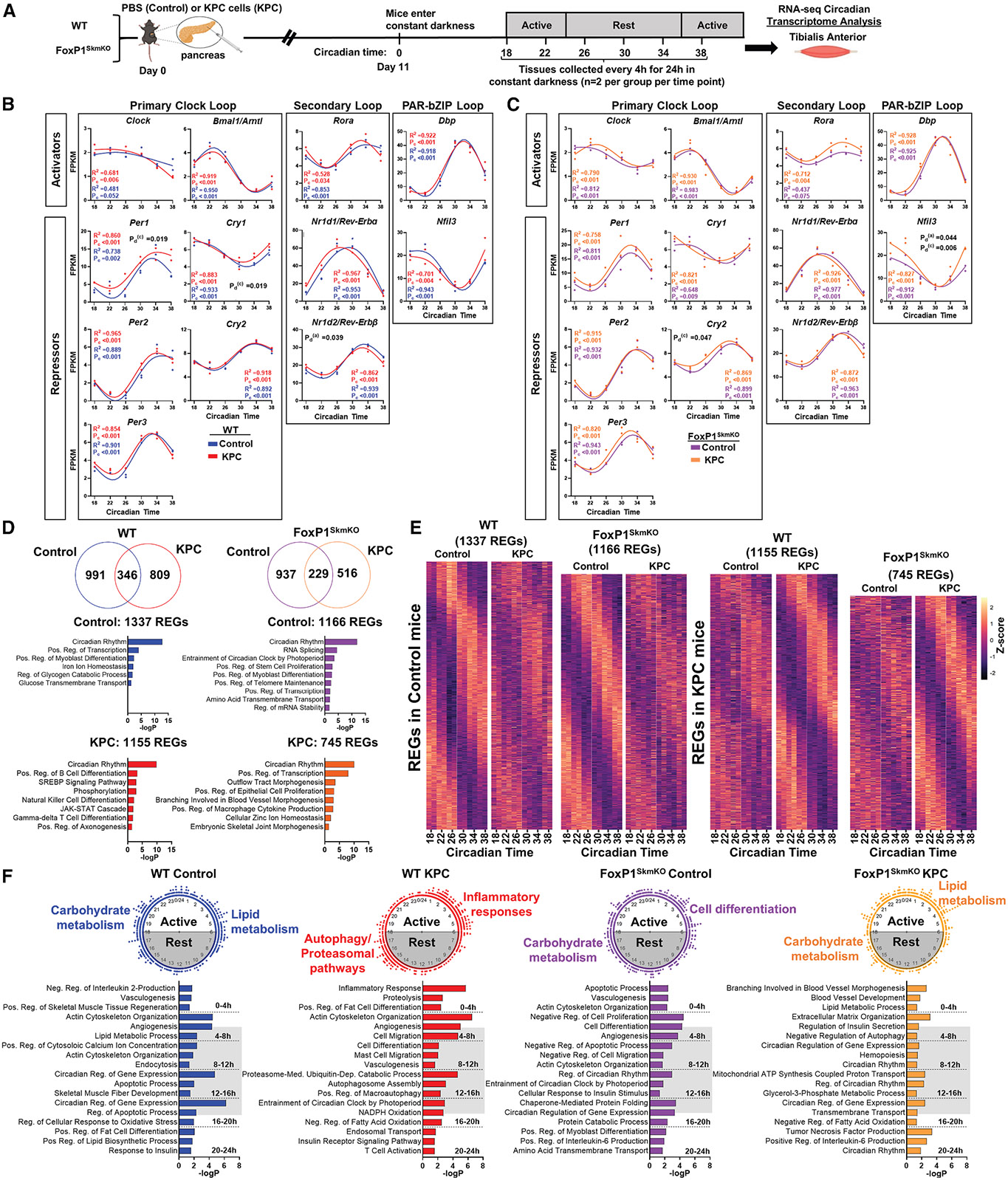
Deletion of FoxP1 within skeletal muscle prevents KPC-induced disruptions to circadian patterns of transcription (A) Simplified study-design schematic. (B and C) Circadian expression levels normalized by fragments per kilobase of exon per million mapped fragments (FPKM) of core clock genes between conditions. Circadian rhythmicity (*p*_c_ < 0.01), and differences in circadian patterns within genotypes for amplitude (*p*_d_^(a)^ < 0.05) and basal expression (*p*_d_^(c)^ < 0.05) were detected across circadian times (CTs) 18, 22, 26, 30, 34, and 38 using R packages LR_Rhythmicity and LR_diff. Goodness of sinusoidal wave fitting (R^2^). (D) Overlap of rhythmically expressed genes (REGs) within wild-type (WT) and skeletal-muscle-specific FoxP1 knockout (FoxP1^SkmKO^) mice between conditions and their top-enriched clusters of GO biological processes. (E) Heatmap of *Z*-scored REGs in each condition. (F) Peak time of expression maps of all REGs and their corresponding GO biological processes (*p* < 0.05). Each dot on the clock represents the time of peak expression for a single REG. *n* = 2 mice/condition/genotype/time point. See also Tables S2, S3, and S7.

Although cancer has been shown to disrupt circadian patterns of gene expression in some peripheral tissues such as liver,^22^ to our knowledge, the impact of cancer on circadian patterns of gene expression in skeletal muscle is currently unknown. We therefore designed experiments to determine whether pancreatic cancer alters circadian patterns of gene expression in skeletal muscle and whether this is mediated through FoxP1. To do this, we orthotopically injected KPC cells, or sterile saline as a control, into the pancreas of WT and FoxP1^SkmKO^ mice. Eleven days later, which corresponds to the onset of muscle wasting in this model in our hands,^14,15^ all mice were placed into constant darkness (circadian time 0 [CT0]) for standardized circadian tissue collection.^23^ Tissue collections began 18 h later (i.e., CT18) and continued every 4 h for 24 h, resulting in a total of six time points (Figure 3A), as previously described.^17^ RNA sequencing (RNA-seq) was then performed from RNA isolated from tibialis anterior (TA) muscles at each time point, for each condition, in both genotypes. To identify the skeletal-muscle circadian transcriptome (i.e., rhythmically expressed genes [REGs]) for each condition within genotype, we applied the sinusoidal model implemented in the LR_rhythmicity package in R, which evaluates the goodness of fit (R^2^) to the model based on the 24-h oscillations in transcript abundance (Table S2).^24^ To identify genes that showed differences in their circadian patterns of gene expression between conditions, we used the LR_diff package in R.^24^

To first determine whether the rhythmicity of core clock genes is disrupted in response to cancer, we extracted genes representing key components of the three interlocking loops of the molecular-clock mechanism. We found that, apart from *Clock* and *Rora,* core clock genes in skeletal muscle displayed circadian rhythmicity in all conditions (i.e., *p* < 0.01) (Figure 3B). However, we found that, in WT but not FoxP1^SkmKO^ mice, KPC tumors increased the basal expression of the negative-limb genes *Per1* (*p* = 0.019) and *Cry1* (*p* = 0.019) and reduced the amplitude of the secondary loop gene and repressor of *Bmal1* and *Nr1d2* (*p* = 0.039). Collectively, these findings demonstrate that, in response to pancreatic cancer, although the expression of clock genes remains rhythmic over 24 h, there is a bias toward the negative arm of the clock, which was mediated by FoxP1.

To further examine the extent to which rhythmically expressed genes (REGs) are disrupted in skeletal muscle in response to cancer, we next assessed the impact of KPC tumors on the circadian expression patterns of REGs, identified in skeletal muscle (Figure 3D and Table S2). Of the 1,337 REGs identified in skeletal muscle of WT cancer-free mice, 991 lost their rhythmicity in response to cancer. Gene Ontology (GO) enrichment analysis of these 991 REGs revealed an enrichment of pathways related to carbohydrate metabolism (e.g., glucose transmembrane transport, regulation of glucose metabolic process, and regulation of carbohydrate metabolic process) as well as lipid metabolism (e.g., fatty acid transport, regulation of fatty acid oxidation, and regulation of lipid metabolic process) (Table S2). Of the 991 REGs that lost rhythmicity in WT KPC mice, 104 remained rhythmic in FoxP1^SkmKO^ KPC mice (Table S2). These 104 REGs included 37 direct FOXP1 targets identified via ChIP-seq, including genes involved in carbohydrate metabolism (e.g., *Ppp1r3c* and *Glul*), lipid metabolism (e.g., *Pltp* and *Rora*), and mitochondrial regulation (e.g., *Ubc* and *Hspa1b*) (Figure 3E; Table S2), supporting FOXP1 as a key player in the transcriptional disruptions of these metabolic networks in muscle during cancer. We also found that 809 genes that were not rhythmic in cancer-free mice became rhythmic in response to cancer. These 809 genes were enriched for GO biological processes of macrophage activation, neutrophil activation, proteasomal protein catabolic process, ubiquitin-dependent protein catabolic process, and regulation of autophagy (Table S2). Of these 809 genes, 733 (90%) required FoxP1 for their *gain* of rhythmicity in response to cancer. These FoxP1-dependent genes included 130 direct FOXP1 targets identified via ChIP-seq, including genes linked to autophagy (e.g., *Gabarap* and *Ddit4*), the proteasome (e.g., *Psmd1* and *Psmd11*), ubiquitin-mediated proteolysis (e.g., *Ube2b, Cul2,* and *Pml*), and inflammation (e.g., *Stat5a/b, Clec2d,* and *Fcer1g*) (Figure 3E and Table S2). Collectively, these data show that cancer significantly disrupts the circadian expression of REGs in skeletal muscle, including a loss of rhythmicity in clock-controlled genes related to metabolic function and a gain in rhythmicity in genes associated with the muscle atrophy phenotype—many of which were dependent on FoxP1.

During normal homeostatic conditions, the peak expression time of REGs is evenly distributed throughout the day, likely reflecting time-of-day-dependent biological functions,^17,25^ which was also demonstrated herein within skeletal muscles of cancer-free control mice. In this regard, in cancer-free mice, pathways related to lipid metabolism (e.g., lipid metabolic process) peak in preparation for the rest phase, while those involved in carbohydrate metabolism (e.g., response to insulin) peak in preparation for the active phase (Figure 3F), which is consistent with previous studies.^17^ However, our data show that, in response to cancer, the even distribution in the peak expression of REGs is lost, with REGs re-clustering into two distinct time points aligning with activity offset (active-to-rest transition; ~CT22) and activity onset (rest-to-active transition; ~CT34). This re-clustering includes glycolytic genes and genes involved in oxidative phosphorylation that no longer peaked in alignment with their natural rest/wake cycles (Table S3). Instead, these clustered peaks were enriched for genes that were not rhythmic in control mice and that annotated to pathways involved in inflammation, proteasome-mediated ubiquitin-dependent catabolism, positive regulation of macroautophagy, and negative regulation of fatty acid oxidation. Importantly, these redistributions in peak REG expression in response to cancer in WT mice were not observed in FoxP1^SkmKO^ mice.

### KPC pancreatic tumors induce time-of-day-dependent alterations in skeletal-muscle gene expression in a FoxP1-dependent manner

**Figure 4.**
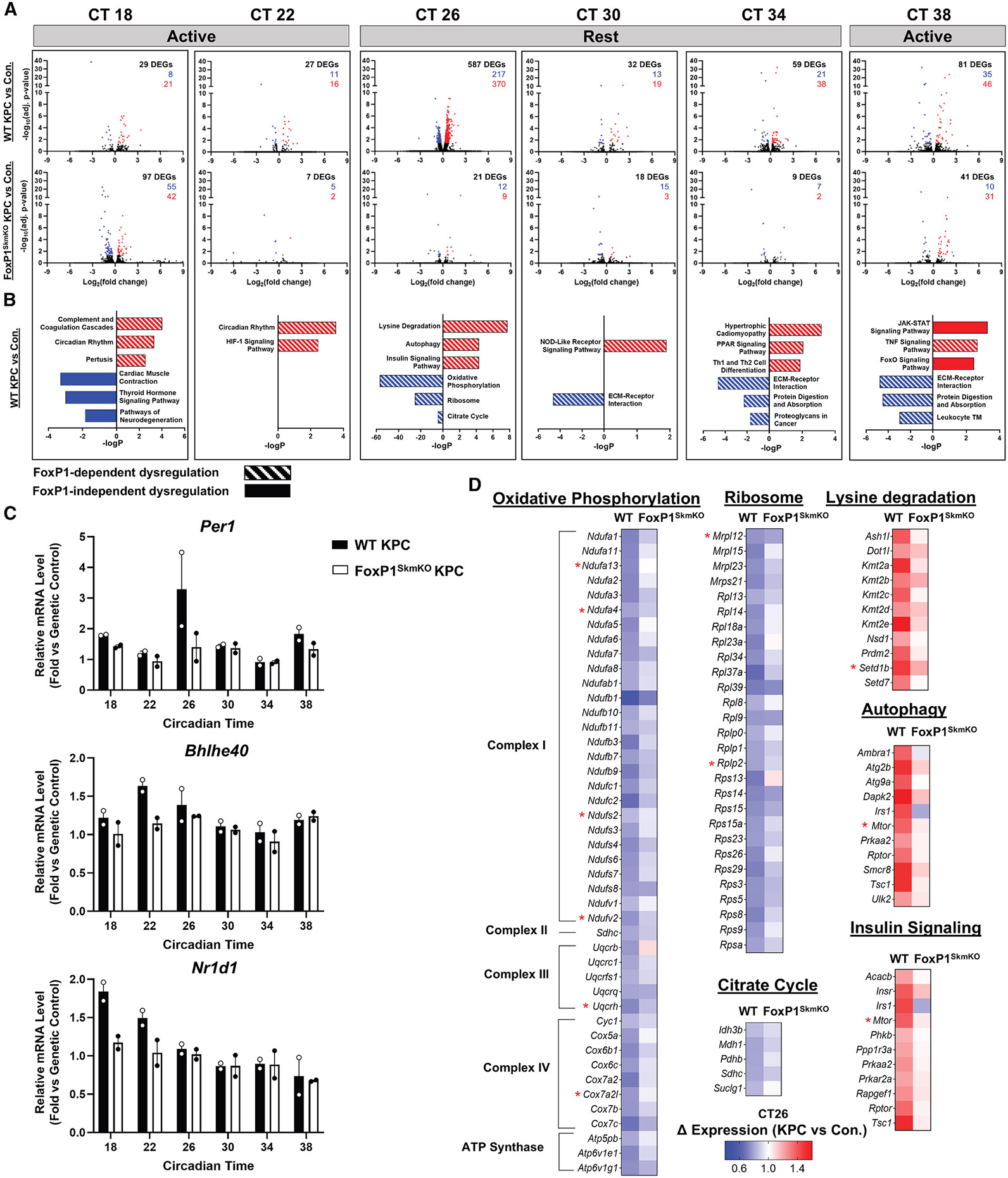
KPC tumors causes time-dependent effects on differentially expressed genes that require FoxP1 (A) Volcano plots of differentially expressed genes (DEGs) (false discovery rate [FDR] q < 0.05) at circadian times (CTs) 18, 22, 26, 30, 34, and 38. (B) Top-enriched upregulated (log_2_ fold change > 0) and downregulated (log_2_ fold change < 0) KEGG pathways (*p* < 0.05) between wild-type (WT) KPC and control mice at each CT. Striped bars indicate FoxP1-dependent dysregulation, while solid bars indicate FoxP1-independent dysregulation in WT KPC mice. (C) Increased expression of core clock genes annotating to circadian rhythm and encoding the negative limb of the circadian mechanism during the active phase (CT18 and CT22) in WT KPC mice compared to cancer-free controls. Data represent individual responses along with mean ± standard error. (D) Heatmaps of FoxP1-dependent DEGs from upregulated and downregulated KEGG pathways at CT26, expressed as fold change relative to corresponding control. Red asterisks highlight FoxP1-dependent genes identified as direct FoxP1 targets with FOXP1-binding sites within their promoter regions through ChIP-seq analysis. *n* = 2 mice/condition/genotype/time point. See also Tables S4 and S5.

To expand our analyses, we separately evaluated differentially expressed genes (DEGs) in skeletal muscle at each of our six time points throughout the day (Figure 4). As shown in Figure 4A and Table S4, there was a time-of-day effect of cancer on DEGs in skeletal muscle of WT mice, with the largest number of DEGs identified following activity offset (CT26). KEGG analysis of genes increased in WT KPC mice compared to cancer-free controls during the active phase (CT18 and CT22) (Figure 4B), identified an enrichment of genes annotating to circadian rhythm, and, specifically, genes encoding the negative limb of the circadian mechanism, including *Per1, Nr1d1,* and *Bhlhe40* (Figure 4C). Pathways of complement and coagulation cascades (e.g., *C3* and *F13a1*) as well as HIF-1 signaling (e.g., *Egln3* and *Pfkfb3*) were also enriched at these time points. Following activity offset (CT26), genes upregulated in KPC mice were enriched for pathways involved in autophagy (e.g., *Ulk2* and *Ambra1*), while downregulated genes were enriched for pathways related to oxidative phosphorylation (e.g., *Ndufa1*, *Sdhc, Uqcrc1, Cyc1, Cox6c,* and *Atp6v1e1*) and the citrate cycle (e.g., *Pdhb, Mdh1,* and *Suclg1*). During the rest phase (CT30 and CT34), ECM-receptor interaction (e.g., *Tnxb, Col4a1,* and *Col6a1*) was the most enriched pathway among genes downregulated in WT KPC mice, while hypertrophic cardiomyopathy (e.g., *Des, Tnnc1,* and *Myl3*) was the most enriched pathway among upregulated genes. Interestingly, following activity onset (CT38), pathways established to be involved in cancer-induced muscle wasting, including JAK-STAT, tumor necrosis factor (TNF), and FoxO signaling pathways, were enriched from genes increased in KPC mice (e.g., *Jak3, Osmr, Nfkbia, Bcl3, Gadd45g,* and *Cebpb*). For each time point, a significant proportion of the genes differentially expressed in response to KPC tumors required FoxP1 (Figure 4B; Table S4). Given that the largest number of DEGs induced by tumor burden occurred following activity offset (CT26), heatmaps of FoxP1-dependent DEGs at CT26 belonging to top-ranking enriched pathways of interest are shown in Figure 4D. Overall, these findings demonstrate that genes annotating to pathways of protein turnover gain rhythmicity in skeletal muscle in response to cancer, showing time-of-day-dependent increases in gene expression, which occurred in a FoxP1-dependent manner. In contrast, metabolic pathways lose rhythmicity in skeletal muscle, with those involved in oxidative metabolism showing time-of-day-dependent downregulation in a FoxP1-dependent manner.

**Figure 5.**
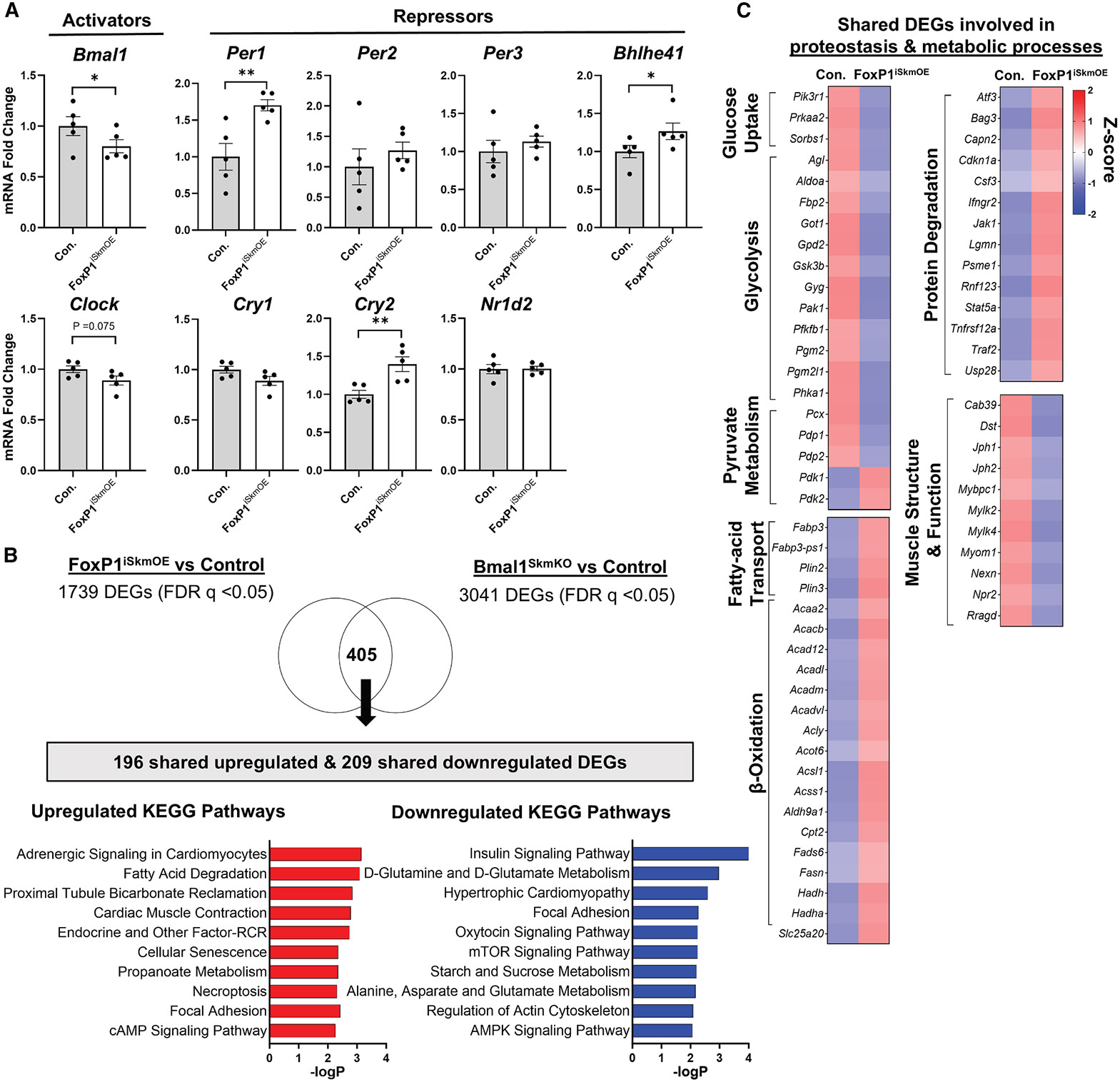
Overexpression of FoxP1 is sufficient to disrupt the expression of core clock and clock-controlled genes within skeletal muscle (A) The effect of inducible skeletal-muscle-specific FoxP1 overexpression (FoxP1^iSkmOE^) on core clock genes extracted from RNA-seq data (*n* = 5 mice/group). Data represent individual responses along with mean ± standard error. Differences between groups were analyzed using unpaired two-tailed t tests; *p < 0.05, **p < 0.01. (B) Overlap of differentially expressed genes (DEGs) (FDR *q* < 0.05) and their top-enriched upregulated (log_2_ fold change >0) and downregulated (log_2_ fold change <0) KEGG pathways from RNA-seq data of skeletal muscle of FoxP1^iSkmOE^ mice relative to control mice and extracted microarray data of mice with a loss of clock function due to skeletal-muscle-specific deletion of *Bmal1* (Bmal1^SkmKO^) and corresponding control mice (*n* = 18 mice/group) (GEO: GSE43071; Dyar et al.^18^). (C) Heatmap of average *Z*-scored shared DEGs involved in proteostasis and metabolic processes in skeletal muscle of FoxP1^iSkmOE^ mice and Bmal1^SkmKO^ mice relative to corresponding control mice. See also Table S6.

FoxP1-dependent DEGs identified above in skeletal muscle of KPC tumor-bearing mice may reflect both direct and indirect targets regulated by FoxP1. To determine which of these genes may be direct FoxP1 targets in the context of tumor burden, we overlapped FoxP1-dependent DEGs at each time point from our circadian RNA-seq dataset with genes showing FoxP1 binding within their promoter region as determined by ChIP-seq (Figure S1; Table S5). Of the 673 FoxP1-dependent DEGs across all time points in skeletal muscle of KPC mice, 108 displayed FoxP1 binding within their promoter regions (74 upregulated and 34 downregulated), supporting these genes as direct FoxP1 targets in response to cancer. Several of the direct FOXP1 targets *upregulated* in response to cancer were transcriptional repressors of the circadian clock, including *Kfl9, Nr1d1, Per1,* and *Bhlhe40,* providing further evidence that FOXP1 is a negative regulator of clock function in skeletal muscle in response to cancer. In line with this, analysis of RNA-seq data collected from skeletal muscles of cancer-free mice with inducible skeletal-muscle-specific FOXP1 overexpression (FoxP1^iSkmOE^) revealed that FOXP1 overexpression is sufficient to upregulate transcriptional repressors of the clock (*Per1, Cry2,* and *Bhlhe41*) and repress clock activators (*Bmal1* and *Clock*) (Figure 5A). In further support of FoxP1 as a negative regulator of clock function in muscle, 23% of genes differentially expressed in muscles of FoxP1^iSkmOE^ mice were found to overlap with genes differentially expressed in muscles with disrupted clock function due to muscle-specific deletion of clock activator, *Bmal1* (Figure 5B).^18^ When considering the function of genes commonly disrupted via either FOXP1 overexpression or *Bmal1* deletion, many are related to pathways known to be disrupted in cancer cachexia.^4,26-28^ In this regard, downregulated genes were commonly enriched for pathways associated with carbohydrate metabolism, including insulin-stimulated glucose uptake (e.g., *Prkaa2* and *Pik3r1*) and glucose oxidation (e.g., *Pdp1* and *Pdp2*), while upregulated genes were commonly enriched for pathways related to lipid metabolism, including fatty acid beta-oxidation (e.g., *Cpt2* and *Hadha*), fatty acid transport (e.g., *Fabp3* and *Plin2*), and protein degradation (e.g., *Psme1* and *Traf2*) (Figure 5C). These findings thus demonstrate that FOXP1 is sufficient to dysregulate clock-controlled genes (i.e., genes regulated by BMAL1).

### Clock transcriptional repressors are negatively associated with muscle size and are increased in skeletal muscle of cachectic cancer patients

**Figure 6.**
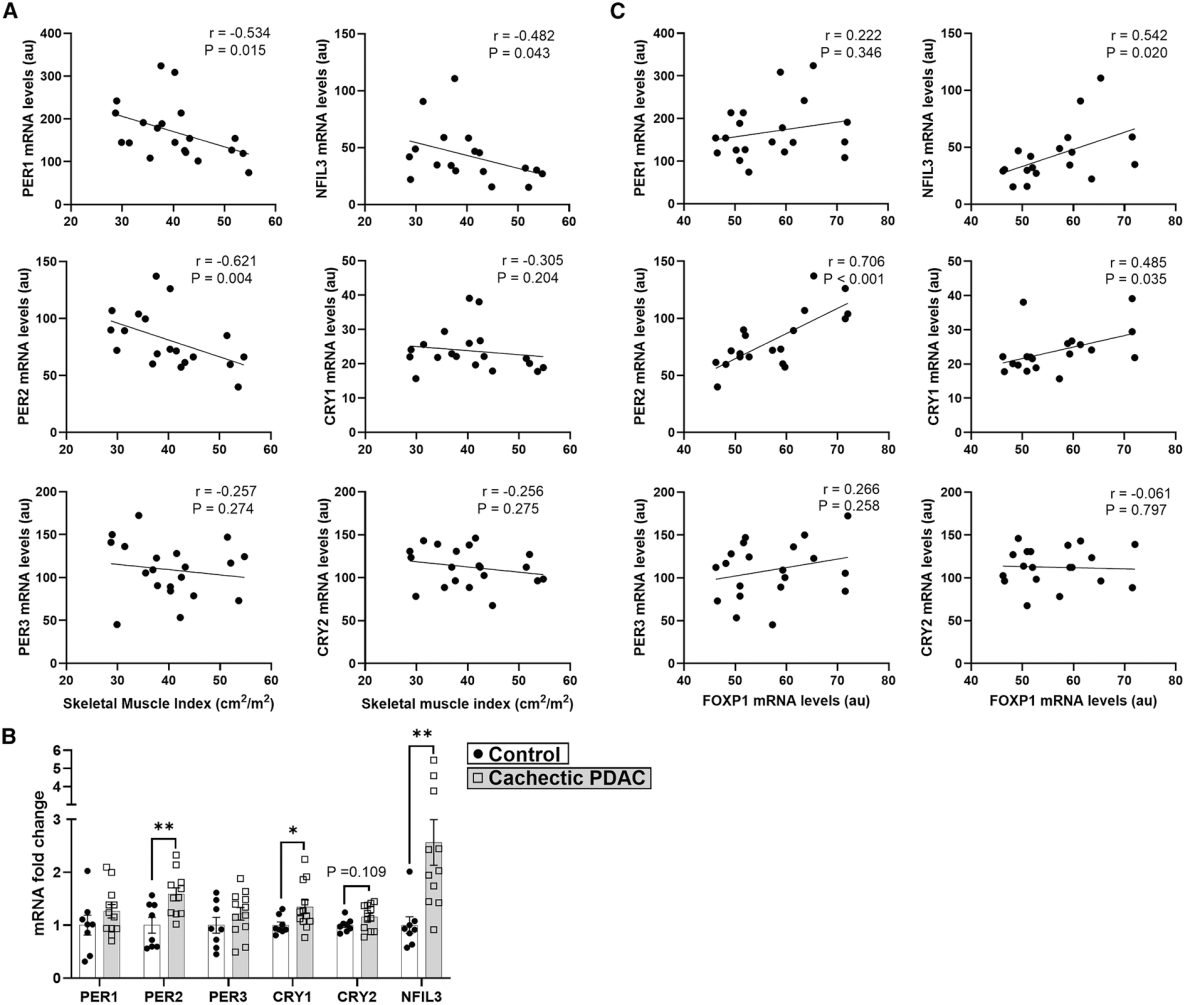
Clock transcriptional repressors are negatively associated with skeletal muscularity and are increased within skeletal muscle of cachectic PDAC patients (A) Correlation analyses for the expression levels of core clock repressors within skeletal muscle of pancreatic ductal adenocarcinoma cancer (PDAC) patients and SMI (*n* = 18–20). (B) Relative expression of core clock repressors in cachectic PDAC patients (*n* = 12) compared to weight-stable non-cancer controls with normal muscularity (*n* = 8). Depending on data normality, tested with Shapiro-Wilk test, comparisons between two groups were analyzed with unpaired two-tailed t tests or Mann-Whitney U tests; **p < 0.01. (C) Correlation analyses for the expression levels of core clock repressors and *FOXP1* expression levels in skeletal muscle of PDAC patients. Data were extracted from microarray analyses of rectus abdominis muscle (GEO: GSE130563) (Judge et al.^26^) and previously published patient SMI data obtained by computed tomography scans (Judge et al.^29^). Correlations were evaluated using simple linear regression analyses. PDAC patient data identified as outliers (ROUT Q = 1%) were excluded from the analyses (*PER2, n* = 1; *CRY1, n* = 1, *NFIL3, n* = 2). Data are presented as individual responses along with mean ± standard error when applicable.

Thus far, our findings indicate that circadian patterns of gene expression are disrupted in cachectic skeletal muscles in response to cancer that may be mediated, at least in part, via FoxP1-dependent upregulation of clock transcriptional repressors. To determine the translational relevance of these findings, we leveraged our previously published transcriptomic dataset obtained from skeletal muscles of patients with pancreatic ductal adenocarcinoma (PDAC).^26^ Core clock genes were extracted and correlated with our published skeletal-muscle index (SMI) data obtained from computed tomography scans of the same patients.^29^ We found statistically significant negative correlations between SMI and expression levels of the clock transcriptional repressors *PER1, PER2,* and *NFIL3* (Figure 6A). We next compared the expression levels of clock genes in muscles from weight-stable control patients, and PDAC patients classified as cachectic based on a body mass loss greater than 5% in combination with low SMI relative to their body mass index (i.e., muscle depletion).^30^ In agreement with our correlation analyses, the clock repressors *PER2, NFIL3,* as well as *CRY1* were significantly increased in skeletal muscles of cachectic PDAC patients compared to weight-stable controls (Figure 6B). Since we identified multiple clock transcriptional repressors as direct targets upregulated by FOXP1 in muscle, we further examined whether *FOXP1* expression levels correlate with the levels of these clock repressors in skeletal muscles of PDAC patients. We found statistically significant positive correlations between expression levels of *FOXP1* and the clock transcriptional repressors *PER2, NFIL3,* and *CRY1* (Figure 6C). These data provide translational relevance to our pre-clinical studies that implicate FOXP1 as an upstream activator of clock transcriptional repressors in skeletal muscle that may contribute to disruptions in circadian patterns of gene expression that are necessary to maintain skeletal-muscle homeostasis.

## DISCUSSION

In recent studies, we demonstrated that the levels of FOXP1 in skeletal muscle of pancreatic cancer patients are tightly linked with cachexia, with high levels of *FOXP1* associated with low skeletal muscularity and increased body weight loss.^12^ Importantly, we further showed that FoxP1 upregulation in skeletal muscle is not only sufficient to drive muscle wasting and weakness but is required for muscle wasting in a murine model of pancreatic cancer.^12,13^ However, the biological processes regulated by FoxP1 in skeletal muscle remained largely unknown. Here, by conducting FoxP1 gain- and loss-of-function studies, we identify skeletal-muscle FoxP1 as a negative regulator of the skeletal-muscle clock that plays a key role in disrupting circadian patterns of gene expression in response to cancer. This is of potential importance given that disruption to the skeletal-muscle clock is sufficient to induce phenotypes common to cancer cachexia,^4,26-28^ including muscle atrophy and weakness, reduced regenerative capacity, fibrosis, and increased insulin resistance.^17-21^ Specifically, we found that FoxP1 directly binds and represses clock activator genes (e.g., *Bmal1*) and activates clock repressor genes (e.g., *Per1, Cry1,* and *Cry2*) and was necessary for the disruptions to circadian patterns of gene expression in skeletal muscle in response to pancreatic cancer. In this regard, we identified that cancer causes a significant loss of rhythmicity in gene programs that normally exhibit circadian expression in skeletal muscle, including those associated with carbohydrate, lipid, and oxidative metabolism, while causing a *gain* in rhythmicity in gene programs linked to muscle wasting, including autophagy, the ubiquitin-proteasome system, and inflammation.

Our findings herein also revealed a substantial interference with the normal temporal distribution of peak expression of clock-controlled genes (i.e., rhythmically expressed genes) in skeletal muscle in response to cancer. These rhythmic oscillating peaks in gene expression are a key component of predictive homeostasis where cellular changes anticipate time-of-day-dependent biological functions in skeletal muscle that are crucial for maintaining cellular and tissue homeostasis. Indeed, these rhythmic changes function to coordinate metabolic pathways and allow adaptations to fluctuations in energy demands during rest/wake and fasting/feeding cycles.^31^ In skeletal muscle, there is an increase in glycolytic metabolism during the wake/feeding cycle and an increase in oxidative metabolism during the rest/fasting cycle.^17^ In preparation for this, circadian predictive homeostasis upregulates the expression of glycolytic genes during rest offset/activity onset as well as the expression of oxidative genes during activity offset/rest onset. However, in the current study, we observed that, in skeletal muscles of mice with pancreatic cancer, many of these metabolic genes lose their rhythmicity and their expression no longer peaks in alignment with their natural rest/wake cycles. Moreover, the expression levels of genes annotating to the citrate cycle and to oxidative phosphorylation pathways in mice with cancer were significantly lower than observed in control mice following transition to the resting phase, when these genes are normally upregulated.^31^ Overall, this results in diminished metabolic flexibility and could compromise the oxidative metabolism of lipids and carbohydrates during a time when the body typically relies on oxidative metabolism, forcing a greater reliance on glycolytic metabolism during this phase. Although circadian metabolomics analyses in muscle are needed to fully understand how cellular metabolism is impacted by tumor burden across the rest/active cycles, a disruption to skeletal-muscle oxidative metabolism is a consistent finding across cachexia studies.^32^

We found that these circadian disruptions to metabolic networks in skeletal muscle are mediated, at least in part, via FoxP1. In this regard, we show that muscle-specific deletion of FoxP1 prevents the cancer-induced loss of rhythmic expression in 104 genes, 37 of which are direct FoxP1 targets and include genes related to carbohydrate metabolism, lipid metabolism, and mitochondrial regulation. FoxP1 deletion normalized their diurnal fluctuations in expression, allowing these genes to peak in alignment with natural rest/wake cycles despite tumor burden. In addition to directly targeting a subset of REGs, our data suggest that FoxP1 may contribute to disruptions in circadian patterns of gene expression through direct binding and regulation of core clock components, most notably transcriptional repressors of the clock. In this regard, we identified several transcriptional repressors of the clock to be increased during the active phase, at CT18 and CT22, in a FoxP1-dependent manner, which would be expected to interfere with the normal rhythmic patterning of genes controlled by the skeletal-muscle clock. However, as mentioned above, a subset of REGs that lost rhythmicity in response to cancer in a FoxP1-dependent manner were direct FoxP1 target genes. Thus, it is possible that FOXP1 bound to gene-regulatory regions in the context of cancer could directly interfere with the rhythmic induction of these genes by the clock transcriptional activators, BMAL/CLOCK. In support of this possibility, our ChIP-seq dataset identified a BMAL1 consensus motif as a top-ranking *de novo* motif within FOXP1-bound DNA regions in skeletal muscle. Moreover, of the 37 genes that lost rhythmicity in response to cancer that were identified as direct FoxP1 targets, 24 (65%) were also identified via ChIP-seq as direct targets bound by BMAL:CLOCK in skeletal muscle (Table S2).^33^ This close proximity in FOXP1 and BMAL1 binding at certain genomic loci could thus lead to co-regulation of these genes, which warrants further investigations.

In addition to mediating the cancer-induced disruptions to the normal rhythmic expression patterns of clock-controlled genes involved in oxidative metabolism, we show herein that skeletal-muscle FoxP1 was required for the gain in rhythmicity of gene programs linked to muscle wasting. Prior studies have consistently shown that genes involved in muscle catabolism are upregulated in the skeletal muscle of tumor-bearing hosts. However, herein, through our analysis of the circadian transcriptome, we add to this existing knowledge base and reveal that genes annotating to pathways of autophagy, proteasomal degradation, and inflammation exhibit time-of-day-dependent increases in their expression in a FoxP1-dependent manner. In the context of cancer cachexia, catabolic pathways involved in muscle protein degradation have been shown to be activated by systemic mediators elevated in the circulation of tumor-bearing hosts.^34^ This includes circulating cytokines that can signal through cytokine receptors present on muscle fibers, as well as cortisol, which is produced following activation of the hypothalamic-pituitary-adrenal axis and can activate atrophy-related genes following binding to the glucocorticoid receptor.^34^ Although speculative, the gain in rhythmicity of genes linked to inflammation and muscle catabolism in response to cancer could therefore be related to circadian programs in other cell types and tissues that govern the expression and secretion of these circulating factors.

In conclusion, our data demonstrate that cancer disrupts the normal circadian patterns of gene expression in skeletal muscle at a time point that we have recently shown coincides with the onset of muscle wasting.^14,15^ We found that, in response to cancer, pathways related to skeletal-muscle wasting become rhythmic, showing increased expression that peaks at activity onset, while pathways involved in carbohydrate, lipid, and oxidative metabolism lose rhythmicity, no longer peaking in alignment with their natural rest/wake cycles. We further show that skeletal-muscle FoxP1 is a key mediator of these disruptions in response to cancer, which may be mediated, at least in part, through direct binding and dysregulation of core clock genes, clock-controlled genes, and genes involved in muscle catabolism.

The identification of circadian disruption in skeletal muscle of tumor-bearing hosts provides an opportunity to study how the clock could be leveraged to attenuate cancer-induced muscle pathologies. In this regard, consistent sleep, feeding, and physical activity patterns have been shown to reset dysregulated circadian rhythms and improve clock function in skeletal muscle and other tissues.^35^ Mechanistically, these lifestyle modifications are thought to improve clock function by enhancing the rhythmic expression of transcription factors, cofactors, and genes that regulate metabolism,^36,37^ and their study in the context of skeletal-muscle clock disruption in tumor-bearing hosts certainly warrants investigation.

### Limitations of the study

As with all studies, there are limitations to this work. The FOXP1 ChIP-seq data were collected from the gastrocnemius-plantaris muscle complex, to provide sufficient starting material, whereas the RNA-seq experiments used the TA muscle, which is commonly used in cancer cachexia studies. Thus, integration of datasets ignores any potential muscle-specific differences. Furthermore, the RNA-seq and ChIP-seq datasets were generated from mice that were 3–4 and 10–12 months old, respectively, and direct target genes of FOXP1 could differ in skeletal muscle based on age. Moreover, we fully recognize that males and females can respond differently to cancer^38^ and that additional circadian studies should be conducted in female mice. We also only studied mice at a time point reflective of cachexia onset, and thus understanding how our findings change with cachexia progression will be important.

## STAR★METHODS

### EXPERIMENTAL MODEL AND SUBJECT DETAILS

All experiments were conducted at the University of Florida and in compliance with the National Institutes of Health Guidelines for Use and Care of Laboratory Animals and approved by the University of Florida Institutional Animal Care and Use. Mice were housed in a temperature-controlled and humidity-controlled facility on a 12-h light/dark cycle, unless otherwise specified, with *ad libitum* access to water and standard diet.

### METHOD DETAILS

#### Cancer cachexia model

Murine pancreatic KPC FC1245 cells, which derive from the tumor of a KPC (LSL-Kras^G12D/+^; LSL-Trp53^R172H/+^; Pdx-1-Cre) mouse, were gifted by Dr. David Tuveson (Cold Spring Harbor Laboratory, Cold Spring Harbor, New York, NY).^46^ The cells were cultured in growth medium (Dulbecco’s Modified Eagle’s Medium with 10% fetal bovine serum and 1% penicillin/streptomycin) in a humidified chamber set to 37°C and 5% CO_2_. The authenticity of the KPC FC1245 cell line has been described previously.^46^ Cell lines were verified to be mycoplasma free prior to receipt and by IDEXX BioAnalytics using PCR evaluation.

Cancer cachexia was induced by orthotopically injecting 2.5 × 10^5^ KPC cells into the pancreas of mice as previously described.^29^ Control littermates underwent a comparable orthotopic surgery, in which the pancreas was surgically exposed and injected with sterile saline. Mice were euthanized 12 days after inoculation which corresponds to the onset of muscle wasting in this model in our hands^14,15^ and before Institutional Animal Care and Use Committee-mandated humane endpoints based on body condition score, body mass loss, and signs of pain and/or distress. Only male mice were included as we recently demonstrated that skeletal muscle expression of FoxP1 is required for muscle wasting and weakness in response to pancreatic cancer in males but not in females.^13^

#### ChIP-sequencing

*Gastrocnemius-plantaris* muscle complexes from the hind limbs were isolated and pooled from 12-week-old C57BL/6J male mice purchased from Jackson Laboratory (Bar Harbor, ME) with and without KPC pancreatic tumors (*n* = 2 mice/group) 2–4 h after lights on at zeitgeber time (ZT) 2–4. Isolated whole muscle samples were sent to Active Motif (Carlsbad, CA) for ChIP-seq. A ChIP reaction was carried out using 15 μg of skeletal muscle chromatin and anti-FoxP1 antibody (Millipore, ABE68, RRID:AB_10631734). ChIP DNA sequences generated by a standard Illumina ChIP-sequencing library were aligned to the mouse genome (mm10). Binding events were called and identified using MACS^16^ with the manufacturer default cutoff of *p* < 1 × 10^−7^. *De novo* motifs were identified via HOMER.^42^

#### Circadian collection and skeletal muscle-specific FoxP1 deletion

Following an acclimation period to ensure entrainment to 12-h light/dark cycle (lights on: 6 a.m.), mice were kept in constant darkness for 18 h (circadian time 0: CT0) in light-tight circadian cabinets (Actimetrics, Wilmette, IL; RRID: SCR 025083) as previously described.^25^ TA muscles were isolated and flash frozen in liquid nitrogen for RNA-sequencing from 39- to 55-week-old male C57BL/6J (WT) mice and homozygous FoxP1^fl/fl^ x ACTA1-Cre (FoxP1^SkmKO^) mice, recently described elsewhere,^13^ with and without murine KPC pancreatic tumors at CT 18, 22, 26, 30, 34, and 38 (*n* = 2 mice/condition/genotype/time point), as previously described.^17^ To maintain constant darkness, mice were euthanized by cervical dislocation under dim red light. We obtained FoxP1^fl/fl^ mice from Dr. Haley Tucker at the University of Texas at Austin^39^ and ACTA1-Cre mice were purchased from The Jackson Laboratory (#006149). To determine differences in core clock gene expression, TA muscles were isolated and flash frozen in liquid nitrogen for qRT-PCR from 20-week-old male WT and FoxP1^SkmKO^ mice with and without murine KPC pancreatic tumors 2–4 h after lights on (6 a.m.) at ZT2-4 (*n* = 4 mice/condition/genotype).

#### Inducible skeletal muscle-specific overexpression of FoxP1

To enable skeletal muscle-specific FoxP1 overexpression (FoxP1^SkmOE^), we obtained FoxP1^flSTOP^ mice with conditional transgenic expression of FoxP1 from Dr. Hui Hu at the University of Alabama^47^ and crossed them with tamoxifen-inducible human skeletal α-actin (HSA)-MerCreMer mice (The Jackson Laboratory, #025750), as previously described.^12^ Tamoxifen was injected intraperitoneally at a concentration of 80 mg/kg of tamoxifen (Thermo Fisher, Waltham, MA) diluted in 100 μL of corn oil on 4 consecutive days to FoxP1^flSTOP^ x HSA-MerCreMer mice to enable overexpression of skeletal muscle FoxP1 (FoxP1^SkmOE^) and to FoxP1^flSTOP^ mice as a control. *Tibialis anterior* (TA) muscles were isolated and flash frozen in liquid nitrogen for RNA-sequencing from 13- to 18-week-old female FoxP1^iSkmOE^ and Control mice (*n* = 5 mice/group). Both groups were euthanized, and muscles were isolated three days following the 4th day of injections 2–4 h after lights on (6 a.m.) at ZT2-4.

#### RNA isolation, qRT-PCR, and sequencing

RNA isolation was performed by homogenization of TA muscles in Trizol (Thermo Fisher) followed by extraction with chloroform, precipitation with isopropanol, and treatment with DNAse (AM1906, Invitrogen). A total of 1 μg of RNA was reverse transcribed using an iScript Advance cDNA Synthesis kit (Bio-Rad, Hercules, CA). Cycle threshold values were measured via quantitative fluorometric PCR (QuantStudio 3, Applied Biosystems, Waltham, MA) on the generated cDNA using TaqMan probes (Thermo Fisher) listed in the key resources table. Gene expression quantification was performed using the 2^−ΔΔCt^ method with *18S* as the reference gene.

RNA sequencing was performed by Novogene (Novogene Co, Ltd., Sacramento, CA). RNA degradation and contamination were evaluated on a 1% agarose gel, purity was checked with a NanoPhotometer spectrophotometer (Implen Inc., Westlake, CA), and integrity and quantity were assessed using the RNA Nano 6000 Assay and Bioanalyzer 2100 system (Agilent Technologies, Santa Clara, CA). A total of 1 μg of RNA per sample was used to generate sequencing libraries. Samples were sequenced on Novogene’s Illumina NovaSeq 6000 (2 × 150bp) to achieve at least 40M reads per sample. Paired-end clean reads were aligned to the reference genome (mm39 for the circadian experiment and mm10 for the FoxP1^iSkmOE^ experiment) using STAR software (v2.5)^44^ and annotated with HTseq-counts (v0.6.1).^45^ Differential expression analyses were performed using the DESeq2 R package (v1.20.0).^43^ The resulting *p*-values were adjusted using the Benjamini and Hochberg’s approach for controlling the false discovery rate (FDR *q*-value).

#### Microarray analyses

Gene expression values in the TA muscle from microarray analyses of WT control mice and mice with skeletal muscle specific *Bmal1* deletion (Bmal1^SkmKO^) from Dyar et al.^18^ were downloaded from NCBI GEO datasets (GSE43071). As previously described, TA muscles were collected from mice (*n* = 3 mice/genotype/time point) at ZT 0, 4, 8, 12, 16, and 20. To evaluate the effect of Bmal1^SkmKO^ on gene expression changes in the TA muscle in the current study, mice were grouped by genotype (*n* = 18 mice/genotype).

To determine translational relevance to individuals with pancreatic cancer, we extracted expression levels of core clock genes of the negative limb from our previously published microarray dataset of *rectus abdominis* muscle biopsies from non-cancer weight-stable control patients (*n* = 8) and patients with PDAC (*n* = 20) (GEO: GSE130563).^26^ In patients, the rectus abdominis was collected during surgery for pancreatic access, which minimized the need for additional patient interventions. Patient SMI data used for correlation analyses in the current study were previously published.^29^

### QUANTIFICATION AND STATISTICAL ANALYSIS

When applicable, data are presented as individual responses and mean ± standard error. In microarray and RNA-seq analyses, genes with an FDR *q*-value <0.05 between conditions were considered differentially expressed. Upregulated (log_2_ fold change >0) or downregulated (log_2_ fold change <0) differentially expressed genes were analyzed separately for their associated functional annotations using EnrichR^40^ or DAVID Bioinformatics database.^41^ Top-ranking non-redundant enriched KEGG pathways or GO Biological Processes were identified using *p* < 0.05. Circadian rhythmicity (*p*_c_ < 0.01) and differential circadian patterns of the transcriptome (*p* < 0.05) were evaluated using R packages, LR_rhythmicity and LR_diff as previously described.^24^ Depending on data normality, tested with Shapiro–Wilk test, comparisons between two groups were analyzed with unpaired two-tailed t-tests or Mann–Whitney U tests. two-way ANOVA with Holm-Sidak post hoc test for multiple comparisons was used to detect differences in core clock gene expression. Correlations were evaluated using simple linear regression analyses. Outliers were examined using the ROUT method with Q = 1%. RStudio (v2024.12.1) and GraphPad Prism (v10.2.3) were used for data analysis, and statistical significance was set to *p* < 0.05 unless otherwise stated.

## Supplementary Material

1

2

3

4

5

6

7

8

Supplemental information can be found online at https://doi.org/10.1016/j.celrep.2025.115689.

## Figures and Tables

**Table:** KEY RESOURCES TABLE

REAGENT or RESOURCE	SOURCE	IDENTIFIER
Antibodies		
Rabbit polyclonal anti-FoxP1	Millipore	Cat# ABE68; RRID:AB_10631734
Chemicals, peptides, and recombinant proteins		
TRIzol reagent	Invitrogen	Cat# 15596018
Deposited data		
FOXP1 ChIP-seq	This paper	GEO: GSE273712
Circadian RNA-seq	This paper	GEO: GSE273878
FoxP1 skeletal muscle overexpression RNA-seq	This paper	GEO: GSE273879
BMAL1:CLOCK ChIP-seq	Gabriel et al.^33^	GEO: GSE143334
Human skeletal muscle microarray	Judge et al.^26^	GEO: GSE130563
Bmal1 skeletal muscle KO microarray	Dyar et al.^18^	GEO: GSE43071
Experimental models: Cell lines		
Pancreatic cancer cells; KPC FC1245 (LSL-Kras^G12D/+^; LSL-Trp53^R172H/+^; Pdx-1-Cre)	Dr. David Tuveson	N/A
Experimental models: Organisms/strains		
Mouse: Wild-type (WT); C57BL/6J	The Jackson Laboratory	Cat# 000664
Mouse: FoxP1^fl/fl^	Dr. Haley Tucker^39^	N/A
Mouse: ACTA1-Cre	The Jackson Laboratory	Cat# 006149
Mouse: FoxP1^flSTOP^	Dr. Hui Hu^12^	N/A
Mouse: HAS-MerCreMer	The Jackson Laboratory	Cat# 025750
Oligonucleotides		
*Clock* (Mm00455950_m1)	Thermo Fisher	Cat# 4331182
*Arntl; Bmal1* (Mm00500223_m1)	Thermo Fisher	Cat# 4331182
*Per1* (Mm00501813_m1)	Thermo Fisher	Cat# 4331182
*Per2* (Mm00478099_m1)	Thermo Fisher	Cat# 4331182
*Per3* (Mm00478120_m1)	Thermo Fisher	Cat# 4331182
*Cry1* (Mm00514392_m1)	Thermo Fisher	Cat# 4331182
Cry2 (Mm01331539_m1)	Thermo Fisher	Cat# 4331182
*Nr1d2* (Mm01310356_g1)	Thermo Fisher	Cat# 4331182
*Bhlhe41* (Mm00470512_m1)	Thermo Fisher	Cat# 4331182
Eukaryotic *18S* rRNA Endogenous Control	Thermo Fisher	Cat# 4448484
Software and algorithms		
Prism	GraphPad	http://www.graphpad.com
EnrichR	Chen et al.^40^	https://maayanlab.cloud/Enrichr
DAVID	Huang et al.^41^	http://david.ncifcrf.gov
GSEA	Gabriel et al.,^33^ Dyar et al.,^18^ Judge et al.^26^	https://www.gsea-msigdb.org
MACS	Zhang et al.^16^	https://github.com/macs3-project/MACS
HOMER	Heinz et al.^42^	http://homer.ucsd.edu/homer
RStudio	R Core Team	https://www.r-project.org
DESeq2	Love et al.^43^	https://www.r-project.org
STAR	Dobin et al.^44^	https://www.r-project.org
HTseq-counts	Putri et al.^45^	https://www.r-project.org
LR_rhythmicity	Ding et al.^24^	https://www.r-project.org
LR_Diff	Ding et al.^24^	https://www.r-project.org

## Highlights

- Cancer causes a loss of rhythmicity in circadian programs in muscle that regulate metabolism
- Inflammatory and catabolic programs gain rhythmicity in muscle in response to cancer
- Skeletal-muscle FoxP1 is required for cancer to induce circadian disruptions in muscle
- Direct targeting of core clock components by cancer-induced FoxP1 may contribute to cachexia
