# Supplementary material for: Cancer-induced FOXP1 disrupts and reprograms skeletal-muscle circadian transcription in cachexia
Source: Cell Rep. Author manuscript; Available in PMC 2025 Jun 14. (PMC12166465; doi:10.1016/j.celrep.2025.115689)
Supplement: 1 [file NIHMS2085659-supplement-1.pdf]

Cell Reports, Volume *44*

Supplemental information

Cancer-induced FOXP1 disrupts and reprograms skeletal-muscle circadian transcription in cachexia

Jeremy B. Ducharme, Daria Neyroud, Martin M. Schonk, Miguel A. Gutierrez-Monreal, Zhiguang Huo, Haley O. Tucker, Karyn A. Esser, Sarah M. Judge, and Andrew R. Judge

A

	**DEGs:** FDR q < 0.05		
	Total # Genes	# Upregulated	# Downregulated
WT, KPC vs Control	735	457	278
FoxP1^{SkmKO}, KPC vs Control	166	76	90
FoxP1-dependent KPC DEGs	673	425	248

B

FoxP1-dependent KPC DEGs with FOXP1 binding sites in their promoter

108 genes (74 upregulated & 34 downregulated)

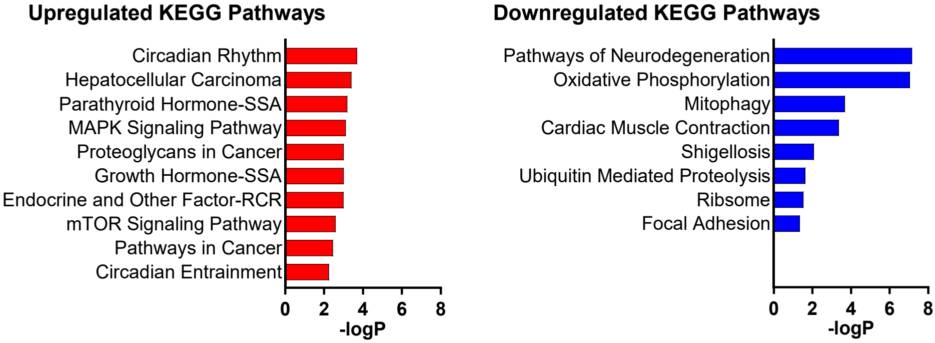

Figure S1. FoxP1-dependent differentially expressed genes with FOXP1 binding sites in their promoter. (A) Total non-redundant differentially expressed genes (DEGs) from our circadian RNA-seq dataset across circadian times 18, 22, 26, 30, 34, and 38 in the tibialis anterior muscle by KPC pancreatic tumor burden relative to controls in mice with either skeletal muscle-specific FoxP1 knockout (FoxP1^{SkmKO}) or their genetic controls (wild-type, WT). Of these, 673 genes were differentially expressed in the tibialis anterior muscle of WT KPC mice but not in FoxP1^{SkmKO} KPC mice and were thus considered as FoxP1-dependent targets. (B) Overlap between FoxP1-dependent DEGs and genes with FOXP1 binding sites in their promoter regions upregulated (log2 fold change > 0) and downregulated (log2 fold change < 0) along with corresponding top-enriched KEGG pathways (n = 2 mice/condition/genotype/time point). See also Table S5.
